# Distinctive modulation of hepcidin in cancer and its therapeutic relevance

**DOI:** 10.3389/fonc.2023.1141603

**Published:** 2023-02-21

**Authors:** Feng Lin, Alex Tuffour, Guijie Hao, Frank Addai Peprah, Aixia Huang, Yang Zhou, Haiqi Zhang

**Affiliations:** ^1^ Key Laboratory of Healthy Freshwater Aquaculture, Ministry of Agriculture, Zhejiang Institute of Freshwater Fisheries, Huzhou, China; ^2^ School of Life Sciences, Jiangsu University, Zhenjiang, Jiangsu, China; ^3^ State Key Laboratory of Bioreactor Engineering, East China University of Science and Technology, Shanghai, China

**Keywords:** hepcidin, cancer, iron, metabolism, therapeutics, homeostasis

## Abstract

Hepcidin, a short peptide synthesized primarily by hepatocytes in response to increased body iron and inflammation, is a crucial iron-regulating factor. Hepcidin regulates intestinal iron absorption and releases iron from macrophages into plasma through a negative iron feedback mechanism. The discovery of hepcidin inspired a torrent of research into iron metabolism and related problems, which have radically altered our understanding of human diseases caused by an excess of iron, an iron deficiency, or an iron disparity. It is critical to decipher how tumor cells manage hepcidin expression for their metabolic requirements because iron is necessary for cell survival, particularly for highly active cells like tumor cells. Studies show that tumor and non-tumor cells express and control hepcidin differently. These variations should be explored to produce potential novel cancer treatments. The ability to regulate hepcidin expression to deprive cancer cells of iron may be a new weapon against cancer cells.

## 1 Introduction

Iron is an element that is necessary for the survival of all living things. Iron is essential for producing enzymes and proteins that catalyze reactions, including cellular respiration, oxygen and electron transport, and DNA synthesis (1). Humans have developed unique transport systems and membrane transporters to preserve iron in a free condition conducive to blood circulation and iron transfer across cell membranes. On the other hand, an excessive amount of soluble redox-active iron might be harmful to cells. Consequently, cells have to carefully adjust the concentration of iron inside their cells to satisfy their metabolic requirements while staying below the level that would cause toxicity. The pro-oxidative effects of iron make excessive iron more harmful, mostly linked to several disorders such as diabetes, cardiovascular disease, liver cirrhosis, cancer and the like (2, 3). Because cancer cells have high iron requirements, iron depletion has been examined for its therapeutic potential. In addition, evidence suggests that modifying one’s iron metabolism may be a viable technique for preventing and treating cancer (4). Iron’s unique importance makes its metabolic and regulatory mechanisms crucial, mainly its physiological and pathological functions. To explain the interplay between anatomically different locations of iron intake, reuse, and use, the idea of controlling iron hormones was put out. In this article, we expound on the differential regulation of hepcidin as a primary regulator of iron balance and elucidate its mechanism in cancer development and its potential therapeutic modalities.

Within the human body, the hormone hepcidin acts as the primary regulator of iron homeostasis. Hepcidin is a long-chain peptide comprised of 25 amino acids (5). Hepcidin induces ferroportin internalization and degradation to control iron absorption and recycling (6). The generation of hepcidin by hepatocytes is significantly boosted by inflammation and iron excess (7). Until 2001, when animal studies showed that iron loading induced hepatic hepcidin mRNA synthesis, there was no evidence that it played a functional role in iron metabolism (8). Hepcidin was discovered in human urine and given the name hepcidin because of its synthesis site (hep-) and antibacterial characteristics *in vitro* (-cidin). In human urine, the most common type is 25 amino acids. However, there are also shorter 22 and 20 amino acid peptides. The core peptide has an easy hairpin structure with a ladder-like orientation, with eight cysteine residues connected by four disulfide bridges. This structure resembles other antimicrobial peptides and is typical of peptides that can damage bacterial membranes (9). This structure is comparable to other antimicrobial peptides and is typical of peptides capable of damaging bacterial membranes (10).

Hepcidin production increases when iron levels are high, but iron released from enterocytes and macrophages decreases. This is in accordance with the hepcidin model, which states that the plasma level of hepcidin mostly governs the rate of iron efflux into the plasma. Likewise, hepcidin synthesis is suppressed as iron levels fall, and these cells produce more iron (11). Hepcidin’s discovery sparked a flood of research into iron metabolism and related problems, fundamentally altering our understanding of human diseases (non-cancerous) caused by excess iron, iron deficiency, or iron disparity (12) (**Table 1**). Cancer and iron homeostasis have been shown to interact occasionally for a substantial period. However, it has only been in recent years that interesting factors into the mechanisms of normal iron regulation have facilitated focused probing of fundamental mechanisms, biological rationale, and pathophysiologic repercussions of alteration in iron metabolism in cancer. There is compelling evidence for the prudent control and distribution of iron in cells and tissues. The finding of the plasma membrane protein ferroportin and the secreted liver protein hepcidin marked the beginning of an essential new era in investigating the relationship between normal iron biology and diseases. As part of the natural process of maintaining iron homeostasis, hepcidin reacts to changes in iron levels and inflammation.

**Table 1:** Effect of hepcidin on iron metabolism and related disorders.

Disorder	Hepcidin expression	Role of hepcidin	Effects of hepcidin	Refs.
Hereditary Hemochromatosis	Decreased	Hepcidin works by inhibiting the release of iron from hepatic reserves, senescent erythrocyte recycling by macrophages, and iron absorption from the gut	Reduced hepcidin transcription as a result of genetically altered BMP/SMAD signaling-related proteins	(13)
Iron loading anemias	Decreased	Hepcidin blocks the entry of iron into the plasma by degrading ferroportin	Reduced hepcidin transcription driven on by anemia, hypoxia, low iron, high EPO, and substances produced from the bone marrow	(14)
Anemia of chronic disease	Increased	Hepcidin limits cellular iron outflow, limiting erythropoiesis iron	Hepcidin transcription is increased as a result of cytokines, ER stress, and bacterial by-products	(15, 16)
Iron -refractory iron anemia	Increased	Hepcidin causes iron shortage and anemia	Genetic deletion of TMPRSS6 causes an increase in hepcidin transcription.	(17–19)
Chronic liver diseases	Decreased	Reduced hepcidin levels cause iron overload, which causes liver iron buildup and non-transferrin-bound iron in the circulation	Oxidative stress results in hepcidin inhibition	(20, 21)
Castleman disease	Increased	The intestinal absorption of iron is inhibited by hepcidin	Hepcidin upregulation brought on by excessive IL-6 production	(22, 23)

**Figure 1:**
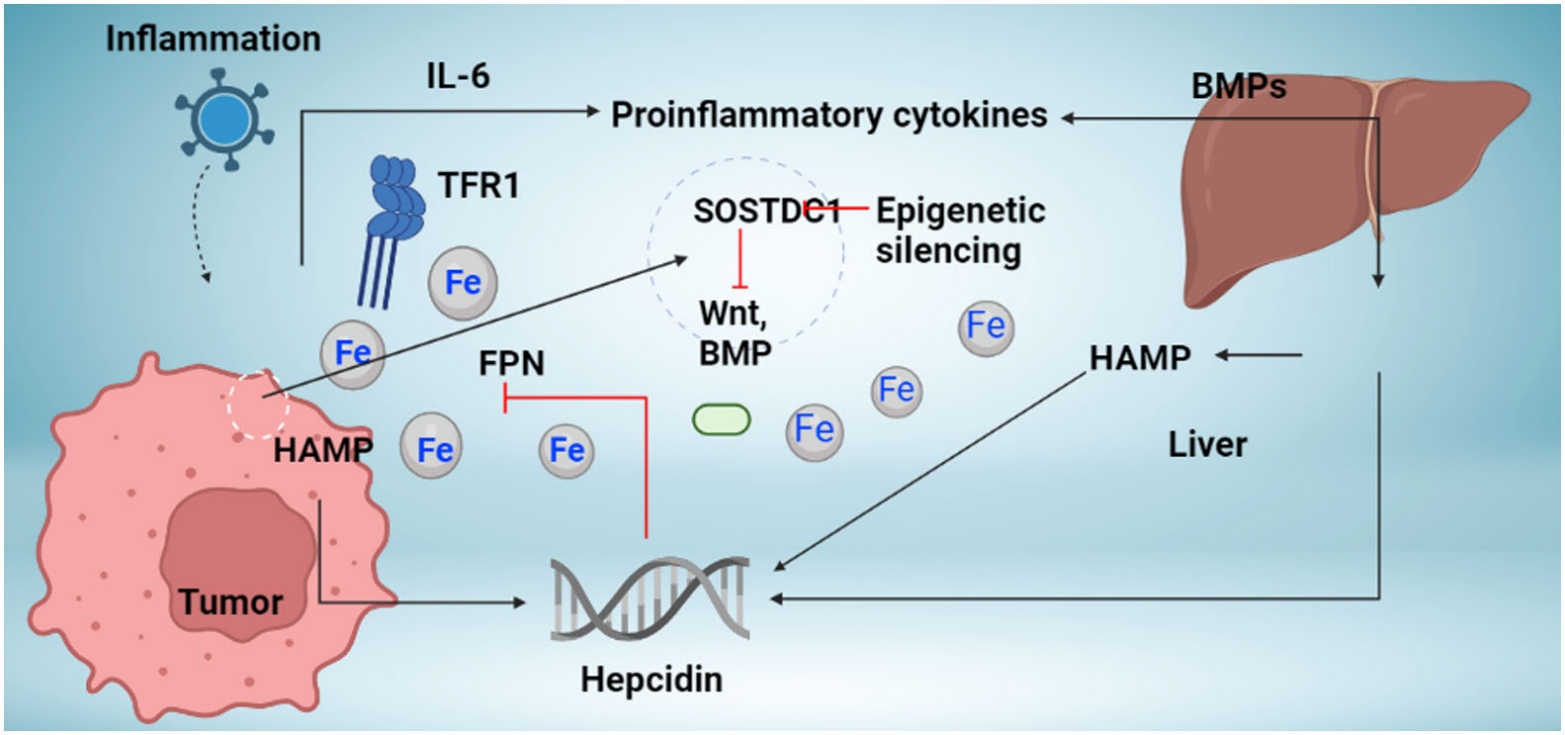
Mechanism regulation hepcidin synthesis in cancer. The liver and local tumor cells in cancer tissue produce hepcidin. Different bone morphogenetic protein (BMP) molecules, including BMP7, and inflammatory triggers, like interleukin-6, are associated with hepcidin overexpression in tumor tissue (IL-6). Studies indicate the presence of novel hepcidin regulators in cancer tissue, including the Wnt pathway and the sclerostin domain-containing protein 1 (SOSTDC1), which is downregulated in cancer due to epigenetic silencing (which is upregulated in cancer). Increased hepcidin in the tumor microenvironment causes ferroportin to behave in a way that causes iron sequestration in tumor cells (FPN). TFR1 overexpression, which increases iron availability to tumor cells, occurs concurrently with an increase in hepcidin. Increased iron depots aid tumor cells in surviving and proliferating.

Inflammatory cytokines like IL-6, bacterial infections, and lipopolysaccharide (LPS) cause transcriptional induction in the hepcidin gene. This connection between inflammatory pathways and hepcidin has a molecular basis for the pathophysiology of chronic illness anemia, a prevalent and, up until now inadequately comprehended side effect of many common medical disorders, including cancer (**Figure 1**). Hepcidin expression in a tumor may be primarily regulated by external and intracellular iron. The results go against the previously established theory for iron homeostasis in cancer cells since cancer cells need to retain iron to proliferate and ingest more iron for development, even though the mechanism causing the decrease of hepcidin mRNA expression in a tumor is still unknown.

## 2 Mechanism of hepcidin activation and expression

### 2.1 Regulation of hepcidin by bone morphogenetic protein (BMP) expression

**Figure 2:**
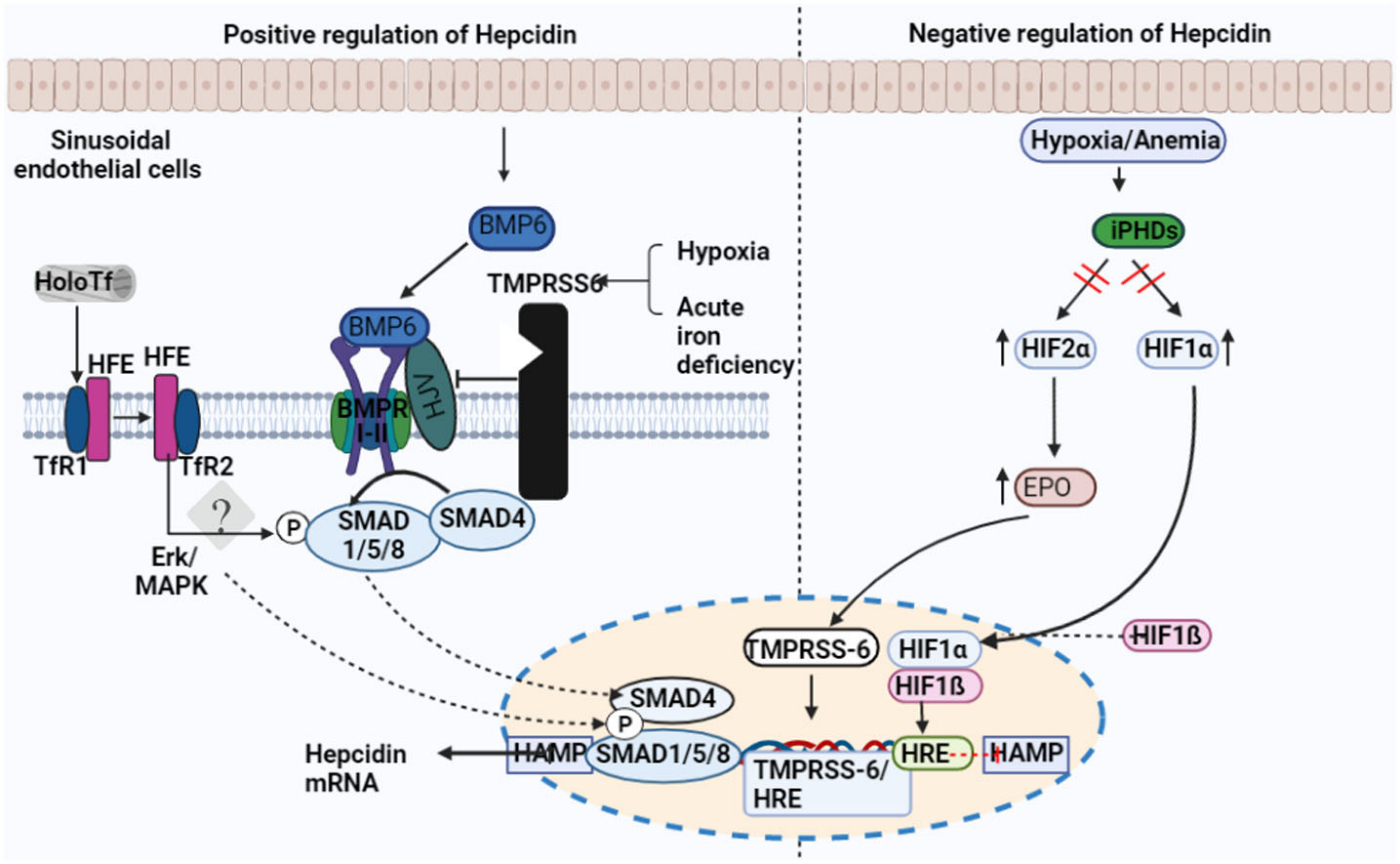
Factors regulating the expression of hepcidin. Transferrin receptor-1 binds Diferric (Holo) transferrin (TfR1). TfR1 induces HFE to interact with cell-surface-stabilized TfR2. Hepcidin transcription is regulated by the interaction between HFE and TfR2. Sinusoid endothelial cells (SEC) and other non-parenchymal cells produce BMP6 when intracellular iron is present. In order to activate BMP6, hemojuvelin (HJV) interacts with BMPRI-II, a receptor for BMP type I and II. The development of the hepatocyte multiprotein complex promotes the phosphorylation of SMAD1/5/8 and their interaction with SMAD4. The nucleus is where SMAD enters to activate hepcidin. The transmembrane serine protease TMPRSS6, which cleaves membrane HJV and suppresses hepcidin mRNA, is made more active by hypoxia and acute iron deficiency.

**Figure 3:**
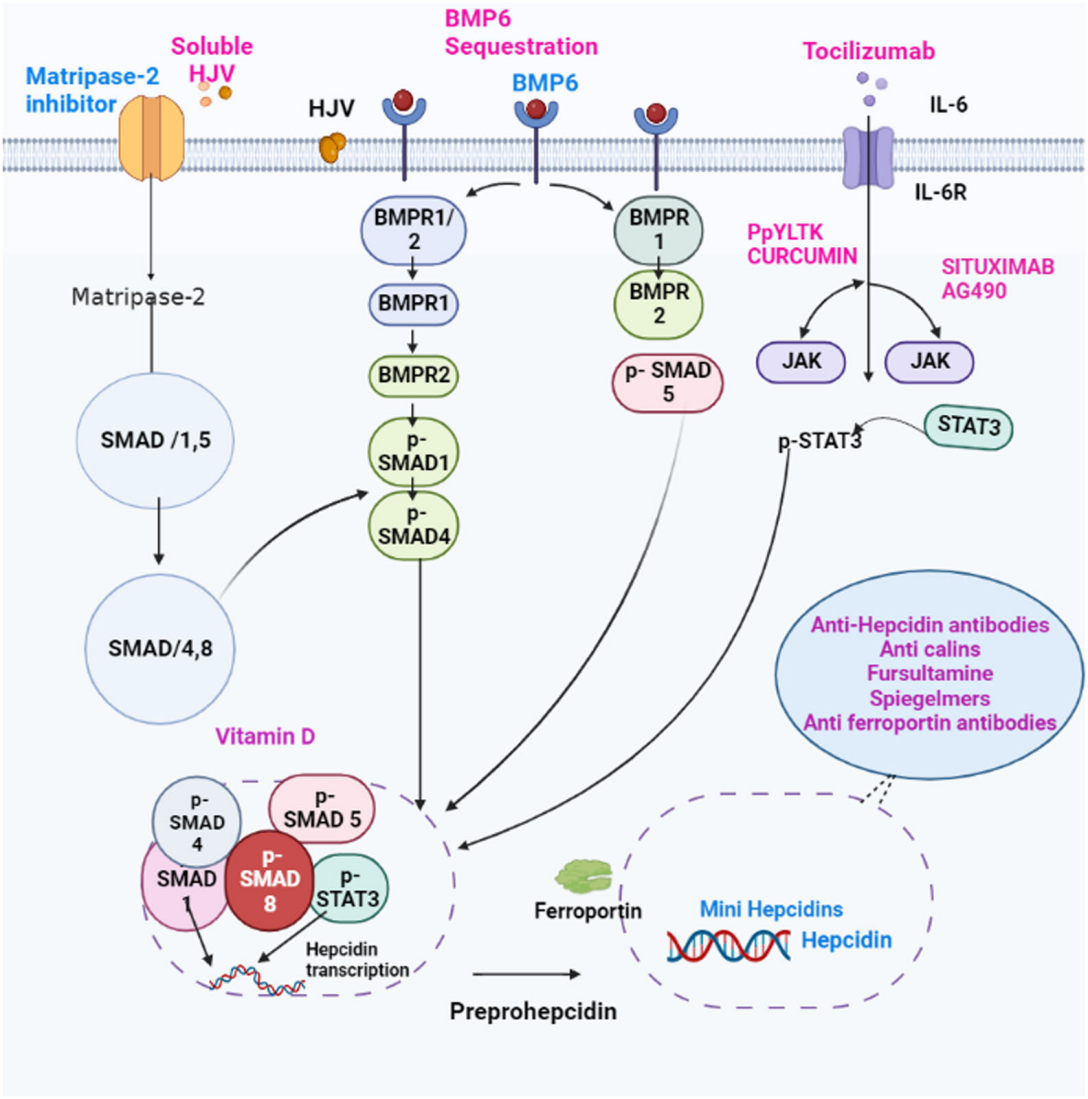
Schematic representation of therapeutic treatments targeting hepcidin. Hepcidin antagonists (Pink). Mediators that inhibit hepcidin expression, anti-hepcidin antibodies, or substances that interfere with the interaction between hepcidin and ferroportin. Hepcidin agonists (Blue) Hepcidin analogues and other agents that boost expression of the protein hepcidin.

The BMP-mothers against the decapentaplegic homolog (SMAD) pathway is essential for controlling iron homeostasis because it controls hepcidin production by modulating transcription (24, 25). Hepcidin signaling, which happens in reaction to increased iron accumulation in the tissue, depends on the proteins BMP2 and BMP6, produced by sinusoidal endothelial cells in the liver. (**Figure 2**) (26, 27). In mice, BMP6 and SMAD 1/5/8 phosphorylation activity were associated with hepcidin mRNA expression, which was regulated by iron status (24). Moreover, it was discovered that BMP6 is a positive regulator for the expression of hepcidin in reaction to iron status and that the lack of BMP6 led to an excess of iron in the body (28). It was finally discovered that BMP6 could actively bind to hemojuvelin (HJV). This protein is found on the membrane of hepatocyte cells and is a component of the iron sensing complex together with the hemochromatosis gene (HFE) and transferrin receptor 2 (TFR2) (25, 28). Activin receptor-like kinase 3 (ALK3) and ALK2 and BMPR2, perhaps in heterodimers with HJV, appear to bind to a receptor complex composed of BMP 2 and 6, which are produced in response to iron. This binding results in SMAD 1/5/8 phosphorylation, which then causes SMAD4 to form a complex with SMAD1. The complex enters the nucleus and attaches to promoters, increasing the production of hepcidin mRNA. (**Figure 3**) (25, 29). However, HJV is rendered inactive by being converted to its soluble form by a serine protease encoded by matriptase-2 (TMPRSS6), which also lowers the transcriptional level of hepcidin and prevents this positive pathway for hepcidin (25, 30). The idea is that BMP2 and 6 are secreted by liver sinusoidal endothelial cells in response to tissue iron overload, whereas TFR1 and TFR2, along with HFE, are the detectors for circulating iron stores estimated through transferrin-iron saturation (27); Others have asserted that BMP2 helps monitor circulating iron while BMP6 serves as a sensor for storing iron.

### 2.2 Regulation of hepcidin by transferrin receptors

TFRs are known to be involved in the expression of hepcidin. The binding of transferrin and internalization of iron that has been coupled to transferrin is carried out by a class of proteins known as TFR (31). Transferrin is coupled to either monoferric or diferric iron that is absorbed by duodenal enterocytes (32). A cell-surface protein called TFR1 facilitates the uptake of iron through receptor-mediated endocytosis. According to recent studies, iron loading may also impact TFR1’s capacity to influence hepatocytes’ ability to express hepcidin (26). Canali et al. determined that hepatocyte-specific ablation of TFR1 resulted in increased hepcidin compared to the liver’s iron level (26). According to their model, the iron deficit increased TFR1, which bound to HFE and inhibited activation of the HFE-TFR2 complex, which prevented the production of hepcidin from being induced (26, 33). TFR2 is similar to TFR1, except it is produced by hepatocytes and is only found in the liver (34). Although some evidence suggests that HFE’s effects on hepcidin expression may be independent of TFR2, it is widely acknowledged that the interaction between TFR1, TFR2, and HFE is crucial for hepcidin expression in response to circulating iron reserves (34, 35). As previously mentioned, the formation of a multiprotein complex by the HFE-TFR2 complex, which activates SMAD 1/5/8 *via* phosphorylation by HJV/BMP, leads to an increase in hepcidin synthesis (HFETFR2-HJV) (36–38).

### 2.3 Downregulation of hepcidin expression by erythropoiesis

Erythropoiesis is a significant physiological mechanism contributing to the downregulation of hepcidin expression (25, 29). The hormone erythroferrone (ERFE), which is released by erythroblasts in reaction to erythropoietin (EPO), appears to be connected to the mechanism because it directly inhibits hepcidin in the liver (**Figure 2**) (39). ERFE mediates hepcidin suppression caused by anemia, erythropoiesis, and blood loss; the postulated mechanism is ERFE binding to BMP ligands (39, 40). According to studies, patients with beta-thalassemia and iron deficiency anemia had higher ERFE levels (41, 42). ERFE is a crucial hormone that directly affects the liver to decrease hepcidin expression in reaction to erythropoietic stimulation, according to a significant body of evidence. This is consistent with a logical physiological. However, additional variables are likely to play, such as decreased iron storage due to increased erythropoietic requirement in the bone marrow. Mechanism to increase iron absorption and release when the body needs to increase erythropoiesis. Hypoxia-inducible factors (HIFs) are transcription factors that control how cells react to low oxygen levels (29, 43). Under hypoxic conditions, HIF undergoes post-translational alteration, which results in its translocation to the nucleus and subsequent binding to a hypoxia-responsive element (43). However, it has also been proposed that erythropoiesis-induced activation of ERFE and platelet-derived growth factor-BB and HIF-1 binding to the TMPRSS-6 promoter, may indirectly mediate the effect of hypoxia on hepcidin production (**Figure 2**) (43, 44).

### 2.4 Hormones and growth factors regulating factors in hepcidin expression

Hepcidin regulation has been linked to several hormones and growth factors. For example, it is commonly known that male hemochromatosis patients have higher body iron reserves than female ones (27, 45). In addition, other variables, such as testosterone-mediated inhibition of hepcidin synthesis, have been implicated, even though the physiological loss of blood that occurs during menstruation will almost surely have a role (46).

In one study, it was hypothesized that 17 β-estradiol would increase hepcidin expression in HepG2 cells. Also, it was demonstrated that 17 β-estradiol would decrease hepcidin production in human liver HuH7 and HepG2 cells (47). According to reports, progesterone increases hepcidin expression *via* progesterone receptor membrane component-1 (PGRMC1) (48). The hepatocyte growth factor and epidermal growth factor, which work *via* the BMP-SMAD pathway, are additional growth factors that have been shown to inhibit hepcidin (49, 50). Hepcidin production in response to infection may be facilitated by activating the toll-like receptor 4 (TLR4) in macrophages (29). Moreover, c-Jun NH_2_-terminal kinases (JNK) bind to the hepcidin promoter due to LPS-induced TLR4 activation in hepatocytes. TLR4 may also stimulate nuclear factor kappa B (NFkB) signaling, a proposed mechanism by which alcohol inhibits the formation of hepcidin and may explain the elevated hepatic iron reserves typically observed in patients with alcohol-associated liver disease (51, 52).

Furthermore, hypoxia is another factor that has been studied to have a suppressing effect on hepcidin expression. The response to hypoxia is regulated by transcription factors called HIFs (43). Due to posttranslational alteration under hypoxic settings, HIF is translocated to the nucleus and binds to hypoxia-responsive components (43). Despite the possibility that the effect of hypoxia on hepcidin expression may be tangentially induced by activation of ERFE and platelet-derived growth factor-BB as a result of erythropoiesis and by HIF-1 binding to the TMPRSS-6 promoter, hepcidin expression is suppressed in the liver by HIF-2 activation through EPO and possibly through direct binding of HIF-1 to the hepcidin promoter (43, 53). Lastly, furin, a processing enzyme often present in the trans-Golgi network, is another mechanism by which hepcidin production is suppressed during hypoxia. Furin breaks down HJV into its soluble form, which then causes BMP6 to be sequestered and hepcidin expression to be suppressed (54).

## 3 Hepcidin and cancer

Anomalies in iron homeostasis triggered by mutations cause cancer in cells or tissues. Hepcidin, a negative regulator of FPN1 is activated in a number of cancers including breast, colon, and prostate (55–57). Hepcidin dysregulation causes iron homeostasis to be disrupted in cancer (**Table 2**). Iron mobilization from enterocytes and macrophages into the circulation is inhibited by high plasma hepcidin levels (contributing to cancer anemia). It can lead to iron buildup in tumor cells by degrading FPN-1, which activates signaling pathways including Wnt (61) and NF-β (62), that promote tumor growth (63). Furthermore, hepcidin has been proven in several experiments to increase tumor growth. According to a study by Schwartz et al., compared to their wild-type littermates, mice lacking hepcidin in the colonic tumor epithelium significantly decreased the number, burden, and size of tumors in a sporadic model of colorectal cancer. On the other hand, mice lacking FPN1 developed intracellular iron buildup and increased the likelihood of tumor development (64). SOSTDC1, a BMP4/7 antagonist also regulates hepcidin synthesis, providing a new path for cellular iron deficiency in the thyroid gland through the E4BP4/G9a/SOSTDC1/hepcidin pathway, which can be reduced hepcidin secretion and thyroid cancer cell proliferation (57). Systemic iron anomalies in cancer patients have been documented in addition to the iron effectors produced by cancer cells (65–67). High serum hepcidin levels are found in several diseases, including prostate cancer, multiple myeloma, breast cancer and non-lymphoma Hodgkin’s disease (68, 69). Cancer cells frequently increase iron input while inhibiting outflow, resulting in iron buildup. However, how they would react to the increased iron instability is unclear.

**Table 2:** Hepcidin role in cancer regulation.

Cancer types	Hepcidin expression	Factors that regulate hepcidin expression in cancer	Effects of hepcidin	Refs.
Breast cancer	Increased	BMP6, IL-6	Breast cancer patients have MT2 downregulation, which can also result in hepcidin overexpression	(12, 58)
Colorectal cancer	Increased	IL-6	The upregulation of ferroportin, which is negatively controlled by hepcidin, promotes the development of colorectal cancer	(12)
Prostate cancer	Increased	BMP4/7, IL-6	Hepcidin serves as an autocrine hormone that promotes the growth of prostate cancer cells by lowering the iron exporter ferroportin on cell surfaces, enhancing intracellular iron retention, and lowering intracellular iron export	(12)
Thyroid cancer	Increased	BMP4/7	Hepcidin promotes the development of cancer by reducing ferroportin expression and increasing iron limitation	(12)
Multiple myeloma	Increased	BMP2/IL-6	BMP 2 and IL-6 stimulate hepcidin in multiple myeloma, which reduces ferroportin expression and causes anemia	(12)
Renal cancer carcinoma	Increased	IL-6, IL-1, TNF-α	BMP signaling is unknown. IL-6 is elevated in serum hepcidin	(12, 59)
Hepatocellular carcinoma	Decreased	BMP 6, IL-6	Hepcidin is correlated with BMP6/IL6 cytokines and cytotoxic immune infiltration in liver cancer tissues	(12, 60)

### 3.1 Hepcidin regulation on hepatocellular carcinoma

The correlation between hepatic iron buildup and hepatocarcinogenesis has been evident over the past decade because of growing evidence involving molecules controlling iron metabolism or iron-related cell death processes like ferroptosis. Therefore, it is critical to understand the liver’s role in iron homeostasis to understand the association between iron and the development of hepatocellular carcinoma (HCC). First, the liver maintains iron homeostasis by releasing iron into circulation for metabolic needs. Additionally, the liver produces proteins that keep iron equilibrium in the body and also store excess iron (70). Numerous iron-driven pathways can cause HCC or carcinogenesis generally. The most significant is the production of reactive oxygen species (ROS) and the ensuing oxidative stress. The impact of “free” iron, which is present when the iron-binding capabilities of plasma transferrin or intracellular iron-storage protein ferritin are exceeded, has led to the theory that oxidative stress is the fundamental cause of hepatotoxicity under iron-excess circumstances. The toxicity of iron in “Fenton-type” reactions, in which iron promotes the transformation of passive H2O2 into extremely reactive HO, is explained by its redox-active characteristics (71). The iron hormone hepcidin, which upholds systemic iron homeostasis, has a role in HCC pathogenesis. HCC patients may have low amounts of hepcidin, in contrast to tumors with higher hepcidin expression. To find molecular targets for diagnosis, prognosis, and treatments, it is therefore, of enormous clinical benefit to examine the regulation and function of hepcidin in HCC. About 90% of all primary liver cancers are hepatocellular carcinomas (HCC). It is an advanced liver disease and one of the leading causes of cancer-related deaths (72).

In response to tissue iron overload and inflammation, BMP6 and IL6 are two of the main stimulators of hepcidin synthesis in hepatocytes (73). The major cell signaling pathway that controls hepatic hepcidin production in response to iron is activated by the BMPs, which act as ligands (74). BMP6 is the potent and prevalent hepcidin inducer in response to elevated iron. Other BMPs, such as BMP9, BMP4, and BMP2, can activate the transcription of hepcidin in primary human hepatocytes, mouse models, and HCC cell lines (75). Notably, BMPs 6 and 4 are upregulated in HCC patients’ livers, and BMP4 and BMP9 are both triggered by hypoxia in HCC tissues where they are highly expressed (76). Therefore, it would seem plausible to anticipate that HCC would express hepcidin more highly due to the high levels of these BMPs. Nevertheless, this contrasts with what is seen in HCC, which mostly has reduced hepcidin expression (77).

Similarly, IL6 is another intriguing factor that deviates from the usual in the setting of hepcidin in HCC. Hepcidin is categorized as an acute-phase protein and is the primary mediator of the acute-phase response, which IL6 mediates (78). Hepcidin is typically brought on by inflammation, specifically IL6 through the JAK-STAT3 pathway and the non-canonical BMP pathway. Higher serum IL6 levels are associated with a higher risk of developing HCC, and higher serum IL6 levels were found in HCC patients. Similarly, the JAK/STAT pathway is constitutively active in HCC, which promotes tumor proliferation, invasion, and metastasis (79). The hepcidin gene itself being downregulated may be one factor causing low levels of hepcidin in HCC. The DNA on the HAMP promoter region is more heavily methylated in HCC tissues. This reduces the production of hepcidin in HCC by suppressing HAMP transcription (80). Interestingly, this downregulation happens despite normal serum iron levels (131.4 ± 23.4 mg/dL) and normal or high ferritin levels (414.4 (328.2-1121) ng/mL) in some HCC patients, as reported in one study (81) or elevated levels of iron, ferritin, and transferrin saturation in the sera of HCC patients compared to control patients in another study (76). Shen et al. demonstrated that the knockdown of HAMP elevated cellular iron levels, migratory cell ability, and proliferation in human liver cancer cell lines. The first discovery was confirmed in mice, where tumor weights were larger in groups with lower Hamp expression than in controls (82).

In summary, hepcidin downregulation can exacerbate HCC pathophysiology and promote cancer growth. One of the HCC diagnostic markers could be hepcidin. Hepcidin is presented as a promising prognostic marker due to its downregulation, which predicts poor patient outcomes. Hepcidin regulation may be a means of improving current HCC therapy approaches and changing HCC pathophysiology.

### 3.2 Regulation of hepcidin expression in breast cancer

Breast cancer is the most prevalent form of cancer affecting women. More than 2.26 million women were diagnosed with breast cancer in 2020. One of the most crucial objectives of biomedical research is to understand the molecular pathways involved in breast cancer incidence and development due to the intricacy of breast cancer studies and their life-threatening relevance (83). Hepcidin significantly influences the growth of breast tumors. Although ferroportin expression is downregulated in breast cancer, its expression in hepcidin is elevated (55). Hepcidin released by breast cancer cells binds to ferroportin, which causes ferroportin breakdown by preventing iron outflow and increasing iron retention (55, 84). In an attempt to decipher the mechanisms that control hepcidin expression in breast cancer cells, Blanchette-Farra et al. presented a new model for tumor-mediated regulation of iron *via* hepcidin by tumor architecture and the microenvironment of breast tumors (83). The authors investigated the potential function of cancer-associated fibroblasts in controlling breast tumor pathways by iron and hepcidin using a 3D cell culture technique. Accordingly, Blanchette-Farra et al. indicated that multiple molecules from the TGF-β superfamily, including BMP and growth differentiation factor-15 (GDF-15), were responsible for the metabolism of hepcidin in breast cancer. They emphasize that GDF-15 expression was substantially correlated with hepcidin levels in patient samples, both at the mRNA and protein levels, indicating a function for GDF-15 in controlling hepcidin in breast tumors (83). Regarding the relationship between the expression of hepcidin and BMPs, they noted that BMPs, specifically BMP6, were crucial mediators for synthesizing hepcidin in breast cancer cells. Additionally, the proliferation of tumor-associated fibroblasts in breast cancer spheroids causes IL-6, one of the most significant pro-inflammatory cytokines to be secreted, further promoting hepcidin (83). More importantly, to reinforce Blanchette’s findings, another study from a clinical standpoint revealed that the combined expression profile of hepcidin and ferroportin serves as a potent predictor for survival following mastectomy for women with breast cancer (55).

### 3.3 Hepcidin regulation in prostate cancer

In men, prostate cancer (PCa) is a form of malignancy with a high incidence of morbidity and mortality (85). Studies have indicated that prostate cancer cells metastasis, proliferation and angiogenesis have all been linked to intracellular iron excess (60). A prostate cancer-specific tumor biomarker is the prostate-specific antigen (PSA), while an indicator of intracellular iron concentration and storage is the soluble transferrin receptor (sTfR). Hepcidin expression can influence PSA and sTfR levels in prostate cancer cells, which can then influence the proliferation of tumor cells (39). Following these considerations, Wang et al. explored how cell proliferation, migration, and apoptosis are related to hepcidin and iron metabolism in prostate cancer cells (86). They assessed prostate cancer cell lines, subjecting them to overexpression and low expression groups, and determined PSA, sTfR, and ferroportin levels. They showed that prostate cancer cells have high hepcidin levels, which regulate cell growth, migration, and death by enhancing intracellular iron transportation (86).

Moreover, the case for iron dysmetabolism in prostate cancer has lately gained further support from a growing body of studies. It is distinguished by various iron-related proteins expressed differently from normal cells (87). Similar to other malignancies, PCa cells require iron to survive. Iron is also required for the activity of enzymes that regulate the transcriptional activity of the androgen receptor, a known PCa promoter (88). Also, iron is required by PCa cells to “reconfigure” internal enzyme activity to boost energy production and extracellular matrix disintegration (89). According to clinical data, iron sequestration in PCa cancer cells is elevated, although it is low in normal cells next to PCa cells (89). Research has shown that senescent prostate cells have higher iron loads due to altered protein expression that controls cellular iron transport (90). Interestingly and incredibly similar to PCa cells, iron dysregulation is seen in experimental models with senescent prostate epithelial cells. It is characterized by the upregulation of TFR1, IRP2, and ferritin, while FPN is upregulated but predominantly localized intracellularly, preventing it from taking part in iron export (90). Senescent cells experience these alterations due to compromised ferritinophagy, which results in increased iron absorption into ferritin. Senescent cells would receive this signal as a deficiency of intracellular iron, enhancing iron import through activation of TFR1. Ferritinophagy is induced chemically to inhibit this process (90). The need for ferritinophagy and its impact on PCa is crucial in investigating this potential disturbance. Tesfay et al. also agree that it increases intracellular iron retention and promotes the survival of prostate cancer cells. Prostatic hepcidin acts as an autocrine hormone by reducing the iron exporter ferroportin on the cell surface (60). To investigate the expression of hepcidin in regulating ferroportin in prostrate cells, they tested expression in prostate cancer cells and non-malignant prostate epithelial cells and also examined cancer cells whose proliferation is either sensitive or insensitive to androgen. They also evaluated the role of prostatic hepcidin in regulating ferroportin in an autocrine fashion.

A significant finding from their work confirms that hepcidin is produced by normal prostate cells and that hepcidin synthesis is significantly increased in prostate cancer cells and tissue. In detail, hepcidin affects both healthy and cancerous prostate cells significantly and functions in an autocrine manner. It enhances metabolically accessible iron, decreases ferroportin levels and maintains viability. Since hepcidin activity is both a source and a local target in prostate cells, this autocrine regulatory axis may support healthy prostate biology. Additionally, these findings show that local synthesis of hepcidin by peripheral tissues also has significant impacts, even though hepcidin has primarily been researched to regulate systemic iron intake and recycling (60).

Research has shown that a distinct regulatory cascade regulates hepcidin synthesis in prostate cancer. A unique confluence of pathways, including BMP4/7, IL6, Wnt, and the dual BMP and Wnt antagonist SOSTDC1, regulates the synthesis of hepcidin in prostate cancer (60, 91). Furthermore, prostate cancer patients exhibit a faster rate of disease progression due to enhanced epigenetic methylation-mediated silencing of SOSTDC1 (60). The findings imply a new connection between iron metabolism and prostate cancer, and prostatic dysregulation of hepcidin encourages the development and progression of prostate cancer.

### 3.4 Hepcidin regulation in lung cancer

Lung cancer is the second most prevalent cancer and the main factor in cancer-related deaths globally (92). Several malignant malignancies, particularly lung cancer, are closely associated with iron dysregulation at both the onset and growth stages. There is growing evidence that iron plays a significant role in lung cancer. Iron is also significant for creating potential lung cancer treatment plans because it participates prominently in numerous types of cell death. IL-6 is said to be increased in lung cancer patients and linked to lung carcinogenesis and poor patient survival (93, 94). It is known to upregulate hepcidin, which reduces iron efflux from cells and results in cancer-related anemia. According to reports, 88% and 62% of non-small cell lung cancer (NSCLC) patients have high ferritin levels and TFR1 expression. In individuals with NSCLC and small-cell lung cancer (SCLC), the serum ferritin level was shown to be increased (95). Research shows that EGFR regulates iron homeostasis by redistributing TFR1, which boosts cellular iron import and encourages the growth of lung cancer. In NSCLC, the expression of membrane TFR1 and iron levels are significantly linked with EGFR activation (96).

Given the close relationship between iron regulation and its influence on lung cancer development, hepcidin evaluation is the key factor to a broader understanding to decipher the mechanism involved. Therefore, fan et al. looked into how hepcidin is engaged in lung cancer metastasis and immune infiltration to understand better its involvement in this process and its molecular regulation (97). They used the Tumor Immune Estimation Resource (TIMER) online database to analyze the mRNA expression of hepcidin in a number of human cancers, and they discovered increased expressions of hepcidin in colon adenocarcinoma, invasive breast carcinoma, esophageal carcinoma, and other cancer cell types compared to their matched normal tissues.

They also discovered that hepcidin mRNA was expressed more strongly in lung adenocarcinoma (LUAD) and lung squamous cell carcinoma (LUSC) tissues than in healthy lung tissues, according to analyses from the gene expression profiling interactive analysis (GEPIA) and UALCAN databases, a web-based tool that provides in-depth analyses of transcriptome data from The Cancer Genome Atlas (TCGA) and MET500 data (97). They specifically found that hepcidin expression was significantly higher in lung cancer tissues when compared to non-tumor tissues. These findings confirmed an earlier study and revealed that hepcidin might act as an oncogene by promoting lung cancer development and spread (98). The authors then considered the hepcidin gene’s prognostic potential because the level of hepcidin expression is tightly linked to lung cancer growth and metastasis. Following the Kaplan-Meier plotter database, they discovered lung cancer patients with increased hepcidin gene expression had worse general and progression-free survival but not post-progression survival (97). This suggests that hepcidin is closely linked to the prognosis of lung cancer. The scientists also examined the link between hepcidin expression and six other invading immune cells, such as CD4^+^T cells, CD8^+^T cells, dendritic cells, neutrophils, and macrophages, to understand better how these immune cells are related to hepcidin expression. A high positive correlation was found between B cells, CD4^+^T cells, macrophages, neutrophils, and dendritic cells but not between hepcidin expression levels and CD8^+^T cells in LUAD (97). Additionally, LUSC immune cell infiltration by all six different kinds was positively and strongly correlated with hepcidin expression. These findings highlight hepcidin’s prominent role in carcinogenesis and suggest that hepcidin may be crucial in controlling immune cell infiltration in lung cancer (97).

### 3.5 Hepcidin regulation in kidney cancer

The kidney is the organ responsible for the reabsorption of iron. In essence, iron that is both transferrin-bound and non-transferrin-bound is capable of entering the glomerular filtrate. The tubular epithelia reabsorb a significant portion of this iron. Numerous transporters, such as the multiligand megalin-cubilin receptor complex, transferrin receptor 1, divalent metal transporter 1, zinc transporter ZIP8, and zinc transporter ZIP14, have been connected to this reuptake (73, 97, 99). In a study, it was noted that mice lacking megalin had significantly higher levels of urine hepcidin without concurrent glomerular filtration impairment (100). Van Swelm et al. validated the megalin-induced reabsorption of hepcidin in the proximal tubules. However, their research suggests that some of the primary hepcidin isoform, hepcidin-25, is degraded into hepcidin-22 and hepcidin-20, which can maintain some of hepcidin-25’s pharmacological effects (101).

Hepcidin expression in the kidney is relatively low compared to the liver, which is known to be the ultimate source of hepcidin (8, 102). Kulaksiz et al. claim that renal hepcidin is primarily found in the connecting tubules and the cortical thick ascending limb of nephron tubules (103). Also, Veuthey et al. hypothesized that the proximal tubule produces prohepcidin. However, it should be noted that the authors of this study measured the immunohistochemical expression of this peptide rather than its mRNA expression, which may indicate that the peptide’s presence inside proximal tubular cells is caused by absorption from the blood filtrate, which contains the peptide’s systemic form (103, 104). However, hepcidin expression is not restricted to tubular cells, as there is evidence that it can also be found in the leukocytes of the renal interstitium when the kidney is inflamed (105). IL-6 and interferon-alpha may cause interstitial renal leukocytes to increase the expression of hepcidin, with interferon-alpha having a more substantial effect on renal monocytes than hepatocytes (106).

Additionally, it has been demonstrated that increased oxidative stress caused by acute kidney injury (AKI) results in an upregulation of renal hepcidin expression (107). Studies have also shown that hepcidin may impact renal iron metabolism separately from ferroportin (101, 108). On the other hand, the BMP6/SMAD pathway may stimulate renal hepcidin, much as it was possible with liver hepcidin (60). Finally, it is worth noting that the ablation of renal BMP6 is associated with gradual renal injury spurred on by a considerable rise in iron load and oxidative stress (109).

Having looked at the expression and regulation of hepcidin in the kidney, it is essential to see how its impact influences cancer development in the kidney. Renal cell carcinoma (RCC) is the most frequent malignant kidney tumor and the second most prevalent urological malignancy (110). Therefore, Kamai et al., interested in investigating whether hepcidin is involved in renal cell carcinoma, examined serum hepcidin-25 and compared hepcidin mRNA expression between RCC tissues and non-neoplastic tissues from the same resected specimens. They further examined the possibility of using hepcidin to assess patients with RCC’s prognosis (111). In their findings, they realized that compared to individuals without metastatic tumors, those with metastatic RCC had serum levels of hepcidin-25 that were higher. Also, hepcidin mRNA expression was higher in metastatic RCCs compared to non-metastatic RCCs. They concluded that poorer overall survival in our RCC patients was associated with increased tumor expression of hepcidin mRNA when assessing patients’ prognoses (111). This study demonstrated that an increased serum level of hepcidin-25 is a marker for the metastatic condition. In contrast, an increased amount of hepcidin mRNA in tumor tissue is linked to the tumor progression of RCC. Similarly, Traeger et al. investigated the viability of serum hepcidin in the quest to find a suitable biomarker to correlate patient’s survival in urinary tract urothelial carcinomas (UUTUC) and RCC as a potential biomarker in UUTUC and RCC (59). They also concluded that serum levels of hepcidin were associated with metastases in both UUTUC and RCC, making serum hepcidin a prognostic marker in RCC and UUTUC (59).

### 3.6 Hepcidin regulation in brain tumors

Brain tumor cells have also been discovered to regulate hepcidin distinctively. Some tumors have low local hepcidin levels, whereas in others, the concentrations are similar to those of healthy brain tissue (112). Distinct cell types in the brain regulate iron metabolism differently. For instance, tumor stem cells in glioblastoma extract iron from the surrounding tissue more efficiently than other tumor cells (113). It is also possible that the distinctive hepcidin regulation in brain tumors is due to the significant roles played by pericytes and glial cells in the modulation of hepcidin in brain tissue (114). In any event, similar to how it functions in other tissues, hepcidin found in brain tissue can inhibit FPN (115). Additionally, it has been demonstrated that this hepcidin-induced action in neurons and vascular endothelial cells is associated with the downregulation of TFR1 and the lowering of transferrin-bound iron from the periphery into the brain (115, 116). Additionally, astrocytes’ intriguing phenomenon of hepcidin-induced downregulation of TFR1 was reported (117). This is crucial because TFR1 overexpression promotes the survival of brain cancer cells. Since there are contradictory data about the effect of hepcidin in lowering the iron burden in brain cells, how these hepcidin-induced effects might influence the brain’s iron metabolism is currently unknown (115). Because studies have demonstrated that reducing the iron availability to brain tumor cells might have significant antitumor effects, it is crucial to understand how hepcidin affects the iron metabolism of these tumors (118, 119).

## 4 Therapeutic implications of hepcidin in cancer

More consideration must be given to measuring hepcidin levels in cancer patients. The dynamics between local and systemic hepcidin levels are probably more important than the absolute serum hepcidin levels. Plasma iron concentrations are primarily regulated by hepcidin. A study by Rivera et al. indicates that within 1 hour after hepcidin injection, mice’s serum iron levels dropped significantly (120). Although hepcidin is quickly degraded from the plasma, the effects of a single dose were felt for up to 72 h. This is likely because it takes time to synthesize enough ferroportin (the hepcidin receptor) again.

Several transcription-inhibition techniques are now being tested to reduce superfluous hepcidin synthesis. Similarly, by disrupting the interaction between hepcidin and ferroportin, hepcidin function can be suppressed (**Figure 3**) (121). Most models suggest that relative to healthy neighboring tissue (122), cancer tissues express more hepcidin and less FPN (60, 84). A high hepcidin/low FPN model associated with high TFR1 expression provides additional evidence that enhanced iron supply is the norm in cancer tissue, promoting cellular proliferation in cancer tissues (58, 122). Cancer cell growth is inhibited by suppressing liver hepcidin, and a similar effect can be obtained by knocking out tumor hepcidin (84). Anti-HJV antibodies can stop hepcidin expression from providing a comparable outcome (123). Anti-hepcidin antibodies may be used as part of a local anti-hepcidin strategy to inhibit the activity of the local hepcidin. Anti-hepcidin antibodies treat prostate cancer to restore FPN expression and stop cancer growth. A different tactic would involve preventing local hepcidin from acting on FPN in cancer cells (60). This might be accomplished by transforming cancer cells’ FPN genes, resulting in an FPN mutant that is resistant to hepcidin’s activities. Hepcidin’s use as a therapeutic target has historically coincided with the production of hepatic hepcidin agonists and antagonists. One strategy to combat hepcidin is to use RNA interference-causing agents like short interfering RNA (124, 125). Hepcidin expression in the liver has been demonstrated to be blocked by small interfering RNAs. Still, no hepcidin-specific small interfering RNA has yet been created that has been shown to be effective in human trials (124, 125). However, some hepcidin antibodies, such as LY2787106, have already been tested in human clinical research to manage cancer-related anemia. Although the preliminary findings indicated that patients tolerated the medication well, the observed efficacy of this treatment was regrettably transient. This was likely because of the homeostatic reparative output of hepcidin, which should be considered when blocking liver hepcidin with particular antibodies (126). Inhibiting the activity of hepcidin expression regulators like BMP molecules, including BMPR, HJV, and MT2 is another aspect of hepcidin antagonism (**Figure 3**) (124).

Additionally, pharmacotherapeutic drugs may be used to manipulate hepcidin. For example, iron-chelation therapy has been proven to improve the anticancer effects of medications like sorafenib (127). Interestingly, sorafenib can inhibit the Ras/MAPK pathway, leading to the induction of hepcidin expression. However, it is yet unknown whether this means that sorafenib’s prohepcidin action enhances its anticancer effects in hepatocellular carcinoma (HCC) (128).

Heparins effectively decrease the expression of hepcidin by acting on the BMP/SMAD pathway (125, 128). Furthermore, heparins can bind a variety of BMPs, which explains how they can decrease hepcidin even in BMP6 knockout models (125, 128, 129). Chemically synthesized heparins with non-anticoagulant characteristics, including SST0001, have demonstrated effectiveness in multiple myeloma (MM) and sarcoma mice by suppressing tumor development alone or in combination with conventional chemotherapy (130). However, the processes underlying SST001’s anticancer effects are complex, and it is currently unclear if they are connected to hepcidin suppression.

The only accepted rationale for the use of hepcidin treatments in cancer up until this point has been anemia of cancer (124, 125). High blood hepcidin levels characterize this disease, which also has low hemoglobin and serum iron levels. The primary source of iron for plasma is hepcidin, which elevates FPN downregulation and cellular iron sequestration in enterocytes and macrophages (123, 126, 131). Hepcidin is the main supply of iron for plasma. The ability of local or systemic hepcidin treatment to affect cancer cell proliferation is uncertain.

## 5 Conclusions and future directions

Although iron is required for the proper functioning of cellular metabolism, it can also produce active oxygen because it is a redox-active metal., which has the potential to be hazardous. As a result, the amount of attention dedicated to investigating iron homeostasis in healthy cells and anomalies in iron metabolism in cancer has increased dramatically over the past few years. While the levels of numerous proteins and the activity of many enzymes are altered in most tumors, cancer cells generally maintain iron metabolism pathways similar to those of normal cells. This shows that altering iron metabolism is essential to maintaining tumor cells.

In cancer cells, elevated iron levels promote the activation of iron-dependent proteins, guard against the adverse effects of excess iron, and achieve “adjusted iron homeostasis” in sync with tumor metabolism. Although the particular mechanism is still not entirely understood, there are still some unanswered problems, such as whether the iron metabolism of different tumor cell types is consistent. How can iron levels be monitored in a way that selectively damages tumor cells while protecting healthy cells?

An essential peptide for maintaining cellular iron homeostasis is hepcidin. This is significant because the amount of iron deposited influences the cellular redox state. One of the harmful factors connected to several diseases is oxidative. Therefore, it should not be surprising that hepcidin dysregulation has been linked to conditions like liver cirrhosis diabetes, and heart disease. A hepcidin imbalance may also be common in cancer, although the involvement of hepcidin in cancer has received less attention. Hepcidin levels are generally higher in cancers, except for HCC and some brain tumors, where they are lower. Hepcidin dysregulation in cancer is significant because it gives tumors the iron they need to survive. The precise linkages between activated leukocytes and tumor cells in terms of the interplay of iron metabolism in the cancer environment will need to be addressed in studies on hepcidin aberrations in cancer. Additionally, research should clarify the precise mechanisms by which malignant cells manipulate the expression of hepcidin and other iron proteins to attack non-cancerous cells. In some malignancies, preventing the activity of local hepcidin can slow tumor growth. For instance, in breast cancer inhibiting liver hepcidin along with local hepcidin suppression has been demonstrated to inhibit tumor proliferation. Hepcidin suppression in MM would be more successful by direct actions on hepcidin, as evidenced by the inconsistent outcomes of blocking hepcidin in MM using anti-IL-6 antibodies. Although these results are quite encouraging, similar research from other tumors will help us understand the function of hepcidin treatment in cancer.

When blocking liver hepcidin, we must exercise caution because doing so may have unintended consequences. Hepcidin levels are low in other malignancies, such as HCC; thus, the anticancer approach aims to augment the levels of hepcidin. The situation is more complicated with brain cancer since many brain tumors have low hepcidin levels, except that this observation does not apply to all brain cancers. Hepcidin expression is also regulated differently by various types of brain cells. The findings that suggest that hepcidin therapy may be significant as a safeguard for brain tissue seem to be compatible with the minuscule quantities of hepcidin found in brain tumors. The ability to stop the formation of brain tumors through hepcidin modification is still an open subject.

## Author contributions

FL and AT wrote the original manuscript. YZ and HZ conceptualized the idea. GH, FP, and AH reviewed and edited the manuscript. All authors contributed to the article and approved the submitted version.
